# Characterization of Flavonoids and Transcripts Involved in Their Biosynthesis in Different Organs of *Cissus rotundifolia* Lam

**DOI:** 10.3390/metabo11110741

**Published:** 2021-10-28

**Authors:** Duncan Kiragu Gichuki, Qingyun Li, Yujun Hou, Yuanshuang Liu, Mengxue Ma, Huimin Zhou, Chen Xu, Zhenfei Zhu, Lina Wang, Fredrick Mutie Musila, Qingfeng Wang, Haiping Xin

**Affiliations:** 1 Core Botanical Gardens/Wuhan Botanical Garden, Chinese Academy of Sciences, Wuhan 430074, China; gichuki@wbgcas.cn (D.K.G.); lqy565720975@163.com (Q.L.); houyujun18@mails.ucas.ac.cn (Y.H.); liuyuanshuang18@mails.ucas.ac.cn (Y.L.); mmengxue93@163.com (M.M.); zhouhuimin19@mails.ucas.ac.cn (H.Z.); 201930115@hbmy.edu.cn (C.X.); zhuzhenfei16@mails.ucas.ac.cn (Z.Z.); wanglina71@hotmail.com (L.W.); qfwang@wbgcas.cn (Q.W.); 2 Key Laboratory of Plant Germplasm Enhancement and Specialty Agriculture, Wuhan Botanical Garden, Chinese Academy of Sciences, Wuhan 430074, China; 3 Sino-Africa Joint Research Center, Chinese Academy of Sciences, Wuhan 430074, China; 4 University of Chinese Academy of Sciences, Beijing 100049, China; 5 School of Biological and Life Sciences, Technical University of Kenya, Nairobi 52428-00200, Kenya; musila@tukenya.ac.ke

**Keywords:** *Cissus rotundifolia*, flavonoids, metabolites, biosynthesis

## Abstract

*Cissus rotundifolia* Lam. is used as a medicinal herb and vegetable. Flavonoids are the major components for the therapeutic effects. However, flavonoids constituents and expression profiles of related genes in *C. rotundifolia* organs are unknown. Colorimetric assay showed the highest flavonoid concentration in roots compared to the stem and leaf. Widely target-based metabolome analysis allowed tentative identification of 199 compounds in three organs. Flavonols and flavones were the dominant flavonoids subclasses. Among the metabolites, 171 were common in the three organs. Unique accumulation profile was observed in the root while the stem and leaf exhibited relatively similar patterns. In the root, six unique compounds (jaceosidin, licoagrochalcone D, 8-prenylkaempferol, hesperetin 7-O-(6″malonyl) glucoside, aureusidin, apigenin-4′-O-rhamnoside) that are used for medicinal purposes were detected. In total, 18,427 expressed genes were identified from transcriptome of the three organs covering about 60% of annotated genes in *C. rotundifolia* genome. Fourteen gene families, including 52 members involved in the main pathway of flavonoids biosynthesis, were identified. Their expression could be found in at least one organ. Most of the genes were highly expressed in roots compared to other organs, coinciding with the metabolites profile. The findings provide fundamental data for exploration of metabolites biosynthesis in *C. rotundifolia* and diversification of parts used for medicinal purposes.

## 1. Introduction

*Cissus rotundifolia* Lam., a species of Cissus genus within the grape family, native to Africa, is widely used as a vegetable and medicinal herb [1,2,3,4]. As the leaves are rich in proteins, fatty acids, crude fibers, and minerals [5], *C. rotundifolia* is regarded as a probable source of healthy food. Due to its anti-diabetic and anti-parasitic properties, preparations derived from, leaves, stems, or a whole plant of *Cissus rotundifolia* Lam. are used as conventional remedies for diabetes, obesity, malaria, allergies, and bacterial infections [6,7,8].

To further understand its medicinal active ingredients and toxicity, the chemical constituents of *C. rotundifolia*, especially phenolic components, were extracted and studied [9,10]. Said et al. [10] found that methanolic extract of *C. rotundifolia* had significant central and peripheral analgesic effects, and their inhibitory effect on paw edema was better than indomethacin, while the total flavonoids in the extract accounted for 42.5% of the total phenolic content. Flavonoids possess a wide range of pharmaceutical properties, such as anti-tumor, antioxidant, anti-inflammatory, and anti-viral properties, as well as inhibitory properties against blood clots [11,12,13,14,15,16]. In medicinal plants, flavonoids subclasses, such as flavones, have been isolated from variable tissues and their medicinal traits have been reported. In *Dracaena cambodiana* and *Dracaena cochinchinensis*, which are used in Chinese traditional medicine, flavonoids and related metabolites have been isolated, exhibiting antibacterial and growth inhibitory properties [17,18,19]. Additionally, a flavanone isolated from *Bauhinia variegata* Linn. was reported to be effective against human cancer cell lines [20]. Recently, Alqahtani [21] pioneered and characterized three infrequent C-glycosyl flavones in *C. rotundifolia* and determined that 1-O-(4-coumaroyl)-β-D-glucopyranose was chiefly responsible for the glucose uptake stimulation. Therefore, it can be speculated that *C. rotundifolia* flavonoids are among the metabolites leading to their use in the pharmaceutical field. However, to our knowledge, there is limited information available about flavonoids and their biosynthesis process in *C. rotundifolia*.

As a consequence of the wide range of importance in biological systems and the medical field, the flavonoids biosynthesis pathway in plants has been widely explored [22]. Although there are several important features regarding modifications or decorative reactions of flavonoids still unrevealed, the main trunk biosynthesis pathway is, by and large, conserved across plant species [23]. Flavonoids are synthesized from phenylanine in the phenylpropanoid pathway [24,25,26]. This pathway and flavonoids diversification are regulated by different transcription factors, such as MYBs, bHLH (basic helix-loop-helix), WD40 proteins, and WRKYs [27,28], through regulation of expression for genes involved in this metabolic pathway [29], including phenylalanine ammonia-lyase (*PAL*), cinnamate-4-hydroxylase (*C4H*), 4-coumarate: CoA ligase (*4CL*), chalcone synthase (*CHS*)*,* chalcone isomerase (*CHI*), dihydroflavonol 4-reductase (*DFR*)*,* flavonoid 3′-hydroxylase (*F3′H*)*,* isoflavone synthase (*IFS*), flavonoid 3′,5′-hydroxylase (*F3′5′H*)*,* flavonol synthase (*FLS*), anthocyanidin synthase (*ANS*), anthocyanidin reductase (*ANR*)*,* and UDP-glucose: flavonoid 3-O-glucosyltransferase (*UFGT*). Among these genes, the *FLS* gene family (*FLS*1 and *FLS*2), encoding for key branching enzymes, were characterized in *O. caudatum* [30]. Their functionality was reported in activating the conversion of dihydroflavonols to flavonols, as well as in the hydroxylation of flavanones to dihydroflavonols.

The development and integration of contemporary -omic technologies, including proteomics, transcriptomics, and metabolomics have enhanced understanding of metabolites biosynthesis mechanism at the molecular level [31,32]. Metabolomics represents the physiological events at the cellular level through the exploration of cellular metabolites and has been applied in the detection of low molecular weight metabolites, such as flavonoids in model plants, crops, and fruits [33,34,35,36,37]. However, associating the metabolome to the genome is challenging, even in model plants with plentiful genomic resources [38].

Flavonoids are a diverse group of plant secondary metabolites and have been widely characterized. However, molecular characterization of flavonoids in the Cissus genus is limited despite the wide range of medicinal applications of its members. For instance, *C. quandrangularis* is widely used in the treatment of bone fractures and body weight management, *C. hypoglauca* for sore throats, *C. assamica* to neutralize snake venoms, *C. rubiginosa* for anti-diarrhea, and *C. rotundifolia* for blood sugar management [4]. In the current work, through the integration of metabolomics and transcriptomic analysis, elucidation of flavonoids components, associated variations in accumulation and expression of corresponding genes, were explored in three organs (root, leaf, and stem) of *C. rotundifolia*. The expression patterns for flavonoids-related genes were also examined in the organs. This study aims to reveal metabolic variations across organs of *C. rotundifolia* providing a valuable foundation for further exploration of the species and other members in the genus in modern pharmaceuticals.

## 2. Results

### 2.1. Total Flavonoids Content Estimation

**Figure 1:**
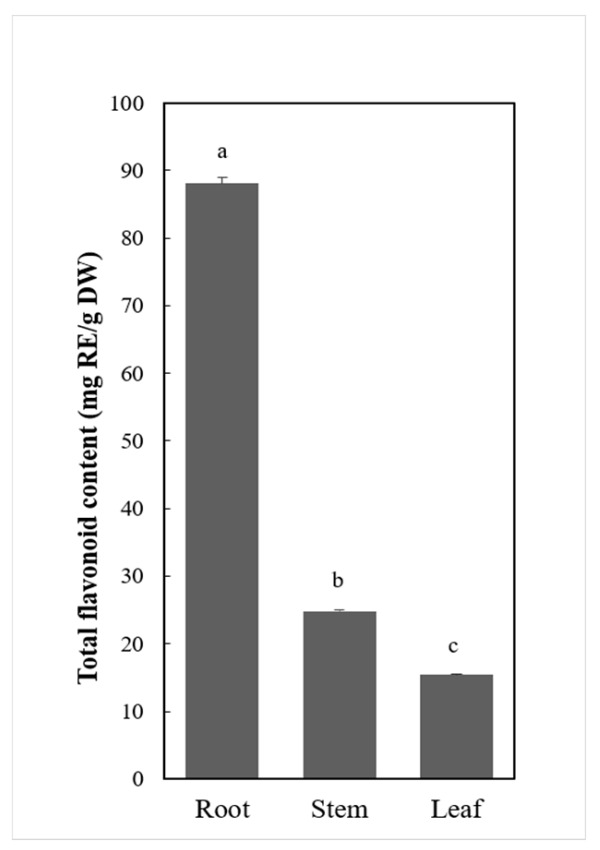
Total flavonoids content for *C. rotundifolia* organs. Flavonoids content was expressed as the rutin equivalent mg/g of the dry weight. The data were expressed as mean ± SD for three replicates. The letters indicate a significant difference at *p* < 0.05.

To determine the accumulation of flavonoids across *C. rotundifolia* organs, the concentration of total flavonoids in leaf, stem, and root was measured by colorimetric methods. The results (Figure 1) showed that root had the highest concentration of total flavonoids, up to 88.11 mg (RE)/g (DW), followed by the stem (24.82 mg (RE)/g (DW) and leaf (15.39 mg (RE)/g (DW). 

### 2.2. Flavonoids Profiling in C. rotundifolia

**Figure 2:**
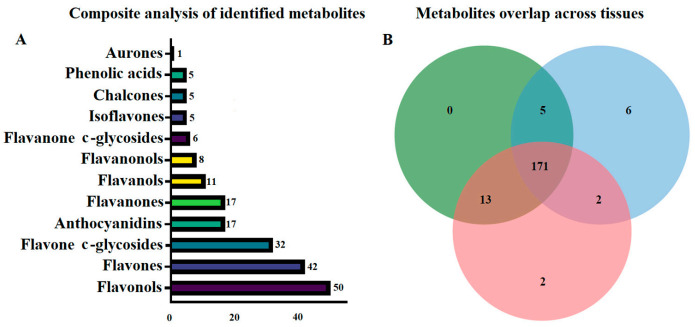
Flavonoids and phenolic acids tentatively identified. (**A**) An integrated view of the identified compounds. The numbers correspond to the compounds in the subclass. (**B**) Metabolites unique or shared among the *C. rotundifolia* organs. The numbers represent the compounds in each section.

**Figure 3:**
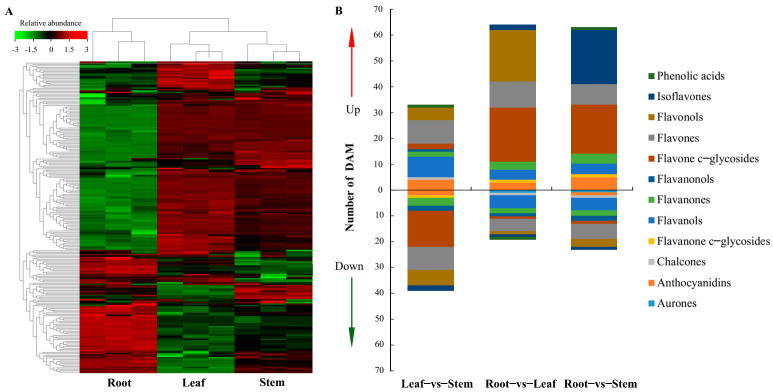
Accumulation profile for tentatively identified compounds. (**A**) Heatmap showing the hierarchical cluster analysis for metabolites identified in *C. rotundifolia* tissues. The colors indicate the metabolites relative abundance with red indicating high values and green for metabolites with lower abundance. (**B**) Stacked bar graph showing the annotation of the identified DAMs between organs. The y-axis represents the number of compounds and the colors indicate the different subclasses.

A total of 199 compounds were tentatively identified in the 3 organs, including 50 flavonols, 42 flavones, 32 flavone c-glycosides, 17 anthocyanidins, 17 flavanones, 11 flavanols, 8 flavanonols, 6 flavanone c-glycosides, 5 isoflavones, 5 chalcones, 1 aurone, and 5 phenolic acids (Figure 2A). Organ-specific identification of the metabolites was also carried out identifying metabolites that were shared across organs as well as those unique to specific organs. In total, 177 metabolites were shared across the three organs with the leaf having no unique metabolite (Figure 2B). In stem, the two unique metabolites were tricetin, and homoeriodictyol. On the other hand, six root-specific compounds were detected, including jaceosidin, licoagrochalcone D, 8-prenylkaempferol, aureusidin, apigenin-4′-o-rhamnoside, and hesperetin-7-o-(6″-malonyl)glucoside. A comparison of metabolites abundance across the organs revealed a unique pattern, in which metabolites with higher abundance in root had variable abundance in stem and leaf tissues. On the other hand, stem and leaf seemingly shared similar patterns in metabolites, abundance (Figure 3A). Details of the detected metabolites including the ion abundance and retention time, are highlighted (Table 1 and Supplementary file S1).

Using Pearson’s correlation coefficient, the repeatability among the intragroup samples was evaluated (Supplementary Figure S1). The rate of contribution of the first two primary principal components was evaluated in PCA. As expected, the three tissues were separated into distinct clusters. The results suggest that standard reproducibility for the tissues and methods used was acceptable allowing further qualitative and quantitative analyses.

### 2.3. Identification of Differentially Accumulated Metabolites (DAMs)

The metabolites with fold change ≥2 or fold change ≤0.5 between different organs were selected. In instances where biological duplication was detected in the sample grouping, metabolites with variable importance in the projection (VIP) ≥1 were selected. VIP value indicates the influence intensity of the difference between the corresponding metabolites in the classification of samples in each group in the model. Generally speaking, the metabolites with VIP ≥ −1 were significantly different. The comparison was carried out across the three *C. rotundifolia* organs. 

In total 72, 83, and 86 differentially accumulated metabolites were identified for leaf–vs–stem (L–vs–S), root–vs–leaf (R–vs–L), and root–vs–stem (R–vs–S) respectively (Supplementary Figure S2). In L–vs–S DAMs, flavones (18) were the majority followed by flavone c–glycosides (16), and flavonols (11) subclasses. In R-vs-L, flavone c–glycosides (22) followed by 21 flavonols were detected and there were 15 flavones among others. Flavonols (24) and flavone c–glycoside (20) were the subclasses with large representation in root–vs–stem DAMs (Figure 3B). 

### 2.4. Transcript Sequencing and Mapping

Using the Illumina sequencing platform, mRNA libraries were generated for leaf, stem, and root tissues of *C. rotundifolia*. The quality of the reads was checked, eliminating low-quality reads and the adapter sequences. A summary of the sequencing statistics has been highlighted in supplementary Table S1. The Q-20 values averaged about 96%, while the GC content ranged between 44% and 47%. Moreover, read mapping ranged between 84.9–86.6% for leaf, 70–70.5% for root, and 86.5–86.9% for stem (Supplementary Table S2). A total of 18,427 expressed genes were identified from the transcriptome data of the three tissues, which covered about 60% of the total annotated genes in the *C. rotundifolia* genome (from our research group; the *Cissus rotundifolia* genome project was deposited at the National Genomics Data Center (https://ngdc.cncb.ac.cn/; to be released on 1 January 2022, (Accessed on: 25 October 2021)) under the BioProject number PRJCA005006). 

### 2.5. Functional Annotation of Identified Genes

Using the eggNOG platform, functional annotation of identified genes was carried out. A total of 17,282 (93.8%) *C. rotundifolia* transcriptome-expressed genes were annotated to COG, KEGG, and GO functional categories. In total, 48.9% (9010 genes) of all genes were characterized into GO terms in the three main ontologies, with 17,109 GO functional terms (Supplementary Figure S3a). The main classes in the major ontologies are listed in supplementary file S2. 

Based on the clusters of orthologous groups of proteins database (COG), a total of 17,282 *C. rotundifolia* genes were annotated into 25 COG functional groups. Among the 25 COG classes, most of the genes were classified under function unknown (S) (Supplementary Figure S3b). As was suggested by Galperin et al. [39], the category for uncharacterized proteins (S) is an important indicator of the progress in the integration of experimental characterization and digital profiling of protein families. To identify the active biochemical pathways in *C. rotundifolia* and improve the understanding of biological functions and gene interactions, KEGG analysis was carried out. A total of 7452 genes were assigned to 233 KEGG pathways, including metabolism (A091000), which was the dominant pathway (Supplementary file S3).

### 2.6. Candidate Genes Involved in Flavonoids Biosynthesis

**Figure 4:**
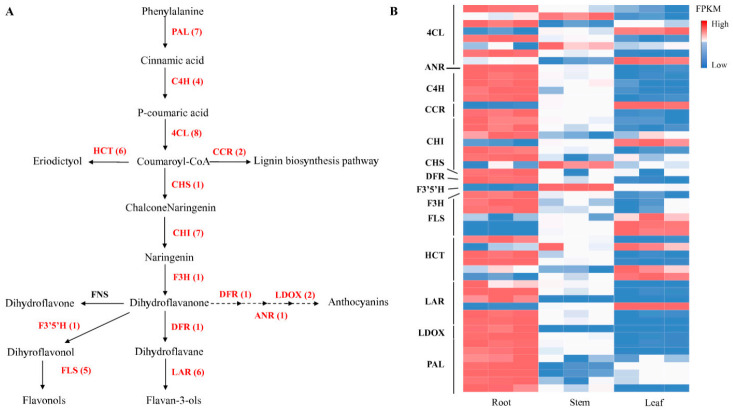
A schematic representation of phenylpropanoid and flavonoids biosynthesis in *C. rotundifolia. *(**A**) Proposed biosynthetic pathway modified from literature and the KEGG pathway database. The numbers in the brackets after each gene name denotes the number of the respective genes in *C. rotundifolia*. (**B**) Heat map representation for flavonoids biosynthetic-related gene expression patterns. *PAL*: phenylalanine ammonia-lyase; *C4H:* cinnamic acid 4-hydroxylase; *4CL:* 4-Coumaric acid: CoA ligase; *CCR:* cinnamoyl-CoA; *HCT: *shikimate-o-hydroxycinnamoyltransferase; *CHS: *chalcone synthase; *CHI: *chalcone isomerase; *F3H: *flavanone 3-hydroxylase; *F3′5′H: *flavonoid 3′,5′-hydroxylase; *FLS: *flavonol synthase; *DFR: *dihydroflavanol 4-reductase; *LDOX/ANS: *leucoanthocyanidin dioxygenase/anthocyanidin synthase; *ANR*: anthocyanidin reductase; *LAR: *leucoanthocyanidin reductase; *FNS: *flavone synthase.

An analysis of *C. rotundifolia* transcriptome revealed multiple transcripts that have been identified to encode enzymes involved in flavonoids metabolism. Most of these genes have higher expression in root compared to leaf and stem (Figure 4B), which is consistent with total flavonoids content. A brief schematic chart was developed (Figure 4A) using the KEGG database and modifying the flavonoids biosynthesis pathway previously described [40]. Generally, the initial process of flavonoids metabolism involves the conversion of phenylalanine through coumaroyl-CoA to chalcones/naringenin by the activation of several enzymes that include phenylalanine ammonia-lyase (*PAL*), cinnamate 4-hydroxylase (*C4H*), 4-coumarate CoA ligase (*4CL*), and chalcone synthase (*CHS*) through the phenylpropanoid pathway. Through the action of shikimate o-hydroxycinnamoyltransferase (*HCT*), among other enzymes, coumaroyl-CoA can also be converted to eriodictyol. Another enzyme, chalcone isomerase (*CHI*), catalyzes the cyclization of chalcone naringenin to naringenins or flavanones, while flavanone 3-hydroxylase (*F3H*), flavonoid 3′-hydroxylase (*F3′H*), and flavonoid 3′5′-hydroxylase (*F3′5′H*) are involved in the hydroxylation of flavanones to various flavonol classes. Additionally, flavonol synthase (FLS) also plays a key role in the conversion of flavanones to the respective flavonols. The number of key genes encoding for the identified enzymes has been highlighted in Table 2 and compared with those identified in *D. cambodiana*. Similar to *D. cambodiana* [40], no transcripts encoding for flavone synthase (*FNS*) were detected from our RNA sequencing analysis.

In addition to the phenylalanine branch of flavonoids biosynthesis, an anthocyanin branch through the action of dihydroflavonol 4-reductase (*DFR*) catalyzes the conversion of both dihydroquercetins and dihydromyricetin to either leucocyanidins or leucodelphinidins. In our study, enzymes that are involved in this branch of biosynthesis were identified and include dihydroflavanol 4-reductase (*DFR*), anthocyanidin reductase (*ANR*), and leucoanthocyanidin dioxygenase (*ANS/LDOX*).

### 2.7. Validation of RNA-Seq Data Using qPCR

The results from RNA-seq were validated using qPCR to determine the correlation in expression from the two techniques. In this study, 12 genes involved in flavonoid biosynthesis were selected for validation (Supplementary Table S3). The primers used were designed using NCBI Primer-BLAST (https://www.ncbi.nlm.nih.gov/tools/primer-blast/ (Accessed on: 25 October 2021)). The primer details have been highlighted in supplementary Table S4. The qPCR analysis was performed using a 7500 Fast Real-Time PCR system (Applied Biosystems, Waltham, MA, USA) in a total of 10 µL reaction volume. The Ct value was determined using the instrument’s software, and the relative quantification of gene expression was monitored after normalization using actin (CRGY0219113) as the internal standard. A comparison of the genes expression patterns obtained from RNA-seq and qPCR was carried out, and the reliability of the RNA-seq was confirmed by the consistency in the expression trends detected by the two data sets (Supplementary Figure S4).

### 2.8. Candidate Transcription Factors Related to Flavonoids Biosynthesis

Several enzymatic and regulatory proteins related to flavonoids biosynthesis were identified and characterized. They included MYB proteins that were involved in the earlier steps in the pathway, regulating flavonol biosynthesis. In the late stages of the pathway leading to the production of anthocyanins and proanthocyanins, a complex of MYB, bHLH, and WD40 proteins (MYB-bHLH-WD40) activated the related genes. Our analysis identified 262 MYB, 70 bHLH, and 169 WD40 protein transcripts that were expressed in the three organs. Their expression profiles were examined, and distinct patterns were observed. Higher expression profiles were observed in roots when compared with both stem and root tissues for the three protein families (Supplementary Figure S5). The expression profiles for the transcription factors exhibited a pattern similar to that of flavonoids and the related genes in the three *C. rotundifolia* organs. 

### 2.9. Differential Gene Expression between Tissues of C. rotundifolia

**Figure 5:**
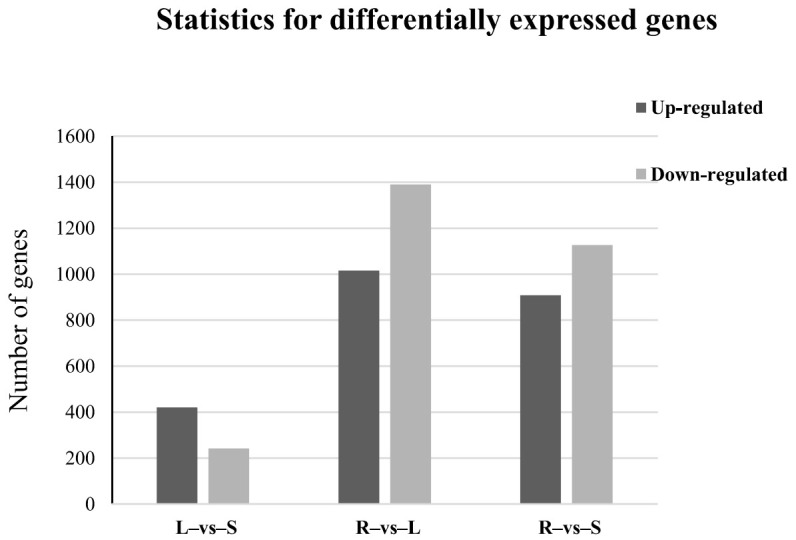
Statistics of differentially expressed genes across three *C. rotundifolia* tissues. R, S, and L represent root, stem, and leaf respectively.

To understand the differences in expression of genes across three organs from *C. rotundifolia*, we used the FPKM (fragments per kb per million fragments) method to digitally profile the expression of genes between leaf, stem, and root [41]. The profiling was carried out for root–vs–stem (R–vs–S), leaf–vs–stem (L–vs–S), and root–vs–leaf (R–vs–L). Differentially expressed genes (DEGs) were reported as those with more than at least a two-fold- change between organs and with a *p*-value ≤ 0.05. Most of the DEGs were identified between root and leaf while a comparison between leaf and stem indicated the least number of DEGs (Figure 5).

### 2.10. GO Enrichment and KEGG Pathway Analysis for Differentially Expressed Genes

The identified DEGs were annotated into the three main GO ontologies. The GO annotation details for the DEGs are highlighted in supplementary Table S5. KEGG analysis for DEGs identified between organs was carried out. Many of the DEGs were associated with metabolic pathways, including secondary metabolites (B09110), which are an important part of medicinal plants (Supplementary Figure S6). 

Functional characterization of DEGs into GO terms and KEGG pathways improved our inference about gene expression patterns across the tissues. Basic functions were the dominant GO terms identified across the board when gene expression levels were compared between organs. Organ-specific KEGG enrichment was observed. DEGs in leaf were annotated into photosynthesis-related pathways, such as photosynthesis and phenylanine metabolism, and carotenoid biosynthesis, among others. DEGs in root were highly enriched in secondary metabolite pathways, including flavonoids (00941), isoflavonoids (00943), and phenylpropanoid (00940), suggesting more flavonoids concentration in the root.

## 3. Discussion

Although *C. rotundifolia* is widely used as traditional medicine, and its extract has also been shown to have anti-diabetic and antioxidant properties, there are few studies on the specific active ingredients of its extract. The extract of *C. rotundifolia* leaves using different solvents, including methanol, acetone, and ethanol, have shown high phenolic content and antioxidant activity [9]. By analyzing the correlation of antioxidant activities and total phenols, Al-Mamary [42] postulated that the antioxidant activities of *C. rotundifolia* were not only related to total phenol content but also related to structures of phenolic compounds and primarily related to their hydroxylation and methylation patterns. Using methanolic extracts from aerial non-flowering tissues of *C. rotundifolia*, Said et al. [43] identified 27 compounds that were dominated by 16 phenolics. Among the identified phenolics, flavanols were the majority. The total flavonoids content from their study using above-ground tissues was 1.35 mg (QE)/g (DW). In our study, the total flavonoids contents were 24.82 mg (RE)/g (DW) and 15.93 mg (RE)/g (DW) for stem and leaf, respectively. The roots had the highest concentration of total flavonoids, suggesting higher potential compared to the other organs. Our results for leaf and stem were in the same range as those reported in *Cissus quadrangularis* and *Cissus adnata *aerial parts [44,45,46]. On the other hand, seven metabolites were identified by Alqahtani et al. [21], including cissoic acid, which belongs to a rare class of secondary metabolites, and cissuxinoside, which they characterized as a new sucrose diester. Further, their study identified and characterized three uncommon C-glycosyl flavones. In this study, 194 flavonoids were tentatively identified in *C. rotundifolia*, which was more than four times the number of metabolites previously identified in leaves [9]. We explored flavonoids in *C. rotundifolia* tissues, including roots that recorded the highest flavonoids concentration and found 8 tissue-specific metabolites (Figure 2B). In general, above-ground tissues are used for medicinal preparation in *C. rotundifolia*, reporting analgesic, anti-ulcerative, and anti-inflammatory properties [10]. However, unique metabolites identified from roots in our study indicated potential use, for example jaceosidin [47] for antidiabetic properties, 8-prenylkaempferol for osteogenesis properties [48], and aureusidin for anti-inflammatory properties, among others. Roots and other underground plant parts have been examined in other medicinal plants and compounds with therapeutic activities reported [49]. Therefore, we postulate that root tissues of *C. rotundifolia* can also be sources for medicinal preparations.

Flavonols, flavones, and flavone C-glycosides were the subclasses with most of the compounds detected in all the organs in this study (Figure 2B). Isoflavones have been associated with pharmacological effects in human medicine, including in weakening menopausal hot flashes [50]. Generally, isoflavones are mainly available in legumes conferring protective roles to plants and nodules. However, from our analysis, five isoflavones with a wide range of pharmaceutical properties, ranging from antioxidants anti-diabetic to anti-mutagenic, were detected, including, calycosin [51], formononetin 7-O-glucoside (Ononin) [52], and pratensein [53]. 

*Cissus rotundifolia* is a valuable medicinal plant. From our analysis, the species is enriched with flavonoids. However, limited information has hindered its exploration at the molecular level. In our study, we provide a transcriptome assembly for *C. rotundifolia* and examine the genes related to flavonoids biosynthesis. Digital expression patterns for candidate genes involved in flavonoids biosynthesis were studied using FPKM (Figure 4B). Flavonoids biosynthesis-related genes showed unique expression patterns in leaves, stems, and roots. 

Anthocyanidins form an important component of metabolites, playing a key role in plants response to abiotic stress, as well as in human health, and anthocyanidin reductase (*ANR*) is a vital enzyme in their biosynthesis [54]. High expression levels for genes encoding for enzymes that participate in the flavonoids biosynthesis, such as *DFR* and *ANS*, have been associated with a higher accumulation of anthocyanins [55]. Our analysis detected two genes encoding for *ANS* and one for *DFR*. The three genes were highly expressed in root compared to other tissues, suggesting their activity in the root, which had the highest flavonoids concentration among the organs. In *Dendrobium officinale*, two *CHS*s were expressed in all of the tissues, but the expressions were especially high in leaves, while five *DFR*s were highly expressed in stems, one in leaves and one in roots, and one of the two *LAR*s was specifically expressed in stems, and the other one was expressed in leaves [56]. In grapevine, the mRNA of *CHS3* accumulated primarily within the berry skin of red cultivars throughout coloration, whereas those of *CHS1* and *CHS2* accumulated within the leaves and berry skin of both the white and red cultivars [57]. 

Through a reaction associated with both *CHS* and *CHI*, the genes encoding these enzymes were reported to be critical in flavonoids biosynthesis and correlated with flavonoids accumulation [58,59]. A single *CHS* encoding gene was identified in our study with the highest expression levels in roots. Moreover, seven *CHI* encoding genes were identified, and their expression levels were generally high in the root tissues. The variation in transcription profile for genes encoding these enzymes across tissues of *C. rotundifolia* could therefore imply their important role in flavonoids metabolism. Additionally, *FLS* is a key enzyme in the conversion of dihydroflavonol to flavonol, thereby associated with the accumulation of flavonols and their composition [60]. Expression levels for *FLS* in our study were variable, with three genes exhibiting higher levels in the leaf while root had two genes had higher expression levels for *FLS* encoding genes. The variable expression levels of *FLS*s across the three tissues could be suggested to have contributed to the higher abundance or the diverse flavonol compounds (Figure 2A). Expression levels of other genes involved in flavonoids biosynthesis have also been linked with flavonoids accumulation levels [61]. From our analysis, no gene encoding *FNS* was detected. This is possibly due to a lack of similarity sequences or due to small-sized transcript fragments, which could not be detected. Generally, from our study, flavonoids biosynthesis-related genes were highly expressed in root and lowly expressed in leaf. The combined expression of genes encoding for these enzymes may partly be associated with the variable concentration of flavonoids and related metabolites in the tissues of *C. rotundifolia*. 

Transcription factors modulate the expression of genes related to biosynthesis, as well as accumulation of secondary metabolites, and are therefore critical in the molecular examination of metabolites accumulation and synthesis [62]. Flavonoids biosynthesis has been reported to be regulated by a complex of transcription factors comprising MYB, bHLH, and WD40 families of transcription factors [63,64]. Examining flavonoids biosynthesis and accumulation across tissues of *Anoectochilus roxburghii*, MYB transcription factors encoding genes were identified and correlated to the observed differential expression and accumulation of flavonoids-related genes [65]. In *E. konishii*, MYB unigenes were identified, and their expression patterns were related to the observed accumulation of metabolites in different tissues. Specifically, consistent expression patterns were reported for a MYB and *FLS* gene, suggesting their role in the observed tissue-specific accumulation of rutin [66]. In our analysis, we identified transcription factor families, including bHLH, MYB, and WD40. In the root tissues, the three transcription factors were generally highly expressed when compared with either leaf or stem tissues (Supplementary Figure S5). It can therefore be suggested that these transcription factors also could have contributed to the observed flavonoids accumulation patterns across tissues of *C. rotundifolia*. 

## 4. Materials and Methods

### 4.1. Plant Materials

Mature stem cuttings of *C. rotundifolia* were collected from Kenya around the Cherangani hills forest reserve. The duplicate voucher specimens SAJIT Z0041 were deposited in the Herbarium of Wuhan Botanical Garden, CAS (HIB) and in the Herbarium of the National museums of Kenya (NMK). The stem cuttings were propagated in pots in the greenhouse for 60 days, with adequate watering and nutrients under natural lighting conditions. Mature tissues were collected, rapidly cleaned, and immediately frozen in liquid nitrogen, after which they were stored at −80 °C until use for metabolome analysis and RNA extraction. For total flavonoids content determination, after cleaning, the samples were sliced into smaller sections and dried in the oven. The dry material was milled and sifted through a 40 mesh filter and stored in tubes for use in total flavonoids content determination. All materials for the study of *C. rotundifolia* were obtained from a single individual main rootstock. Three replicates were collected from each tissue for RNA extraction and metabolomics.

### 4.2. Metabolome

Sample preparation for metabolomics, extraction, identification as well as quantification of the compounds was carried out following conventional procedures developed by Wuhan Metware Biotechnology Co., Ltd. (https://www.metware.cn/ (Accessed on: 25 October 2021)). The repeatability and reliability of the extraction and detection methods were evaluated by analyzing the overlapping of total ion current (TIC) by using quality control samples. The quality control sample was prepared by combining all sample extracts into a combined sample and was injected after every 10 experimental samples. 

#### 4.2.1. Sample Preparation and Extraction

The frozen samples were freeze-dried before grinding using a freeze-dryer (SCIENTZ-100F/A; Ningbo Scientz Biotechnology Co., Ltd. Ningbo, China). Freeze-dried samples were crushed using a mixer mill (MM 400, Retsch) with a zirconia bead for 1.5 min at 30 Hz. In total, 100 mg powder was weighed and extracted overnight at 4 °C with 1.0 mL 70% aqueous methanol. Following centrifugation at 10,000× *g* for 10 min, the extracts were absorbed, (CNWBOND Carbon-GCB SPE Cartridge, 250 mg, 3 mL; ANPEL, Shanghai, China, https://www.anpel.com.cn/ (Accessed on: 25 October 2021)) eluted, and filtrated (SCAA-104, 0.22μm pore size; ANPEL, Shanghai, China, https://www.anpel.com.cn/ (Accessed on: 25 October 2021)) before LC-MS analysis.

#### 4.2.2. HPLC Conditions

The sample extracts were analyzed using a LC-ESI-MS/MS system (HPLC, Shim-pack UFLC SHIMADZU CBM30A system, https://www.shimadzu.com.cn/ MS, Applied Biosystems 4500 Q TRAP, www.appliedbiosystems.com.cn/ (Accessed on: 25 October 2021)). The analytical conditions were as follows: HPLC: column, waters ACQUITY UPLC HSS T3 C_18_ (1.8 µm, 2.1 mm × 100 mm); solvent system, solvent A (water, 0.04% acetic acid) solvent B (acetonitrile, 0.04% acetic acid); gradient program, 100:0 V(A)/V(B) at 0 min, 5:95 V(A)/V(B) at 11.0min, 5:95 V(A)/V(B) at 12.0 min, 95:5 V(A)/V(B) at 12.1 min, 95:5 V(A)/V(B) at 15.0 min; flow rate, 0.40 mL/min; temperature, 40 °C; injection volume: 5 μL. The effluent was alternatively connected to an ESI-triple quadrupole-linear ion trap (Q TRAP)-MS.

#### 4.2.3. ESI-Q TRAP-MS/MS

Linear ion trap (LIT) and triple quadrupole (QQQ) scans were acquired on a triple quadrupole-linear ion trap mass spectrometer (Q TRAP; API 4500 Q TRAP LC/MS/MS System), equipped with an electrospray ionization (ESI) Turbo Ion-Spray interface, operating in a positive and negative ion mode and controlled by Analyst 1.6.3 software (AB Sciex). The ESI source operation parameters were as follows: an ion source, turbo spray; source temperature 550 °C; ion spray voltage (IS) 5500 V; ion source gas I (GSI), gas II (GSII), curtain gas (CUR) was set at 55, 60, and 25.0 psi, respectively; the collision gas (CAD) was high. Instrument tuning and mass calibration were performed with 10 and 100 μmol/L polypropylene glycol solutions in QQQ and LIT modes, respectively. QQQ scans were acquired as multiple reaction monitor (MRM) experiments with collision gas (nitrogen) set to 5 psi. Declustering potential (DP) and collision energy (CE) for individual MRM transitions were done with further DP and CE optimization. A specific set of MRM transitions were monitored for each period according to the metabolites eluted within this period.

#### 4.2.4. Qualitative and Quantitative Analysis of Metabolites

Qualitative analysis of primary and secondary MS data was carried out by comparison of the accurate precursor ions (Q1), product ions (Q3) values, the retention time (RT), and the fragmentation patterns with those obtained by injecting standards using the same conditions if the standards were available (Sigma-Aldrich, St. Louis, MO, USA, http://www.sigmaaldrich.com/united-states.html (Accessed on: 25 October 2021)) or conducted using a self-compiled database MWDB (MetWare biological science and Technology Co., Ltd. Wuhan, China) and publicly available metabolite databases if the standards were unavailable. Repeated signals of K+, Na+, NH4+, and other large molecular weight substances were eliminated during identification. The quantitative analysis of metabolites was based on the multiple reaction monitoring (MRM) mode. In the quadrupole (Q Trap), the precursor ions (parent ions) of the target compound were first selected. To eliminate the interference by non-target substances, the precursor ions were ionized by the collision chamber forming other fragment ions. Fragment ions were screened through the triple quadrupole, to select the specific fragment ion while eliminating the interference of the non-target ions. The characteristic ions of each metabolite were screened through the QQQ mass spectrometer to obtain the signal strengths. Integration and correction of chromatographic peaks for similar metabolites in different samples were performed using MultiQuant version 3.0.2 (AB SCIEX, Concord, ON, Canada). The corresponding relative metabolite contents were represented as chromatographic peak area integrals. The analysis was carried out in triplicates for each set sample.

### 4.3. Determination of Total Flavonoids Content (TFC)

#### 4.3.1. TFC Extraction

Flavonoids extraction was carried out using the methods described by AL-Bukhaiti et al. [9] for phenolic extraction with minor modifications. The method is based on the spectrometric determination of the complex formed when flavonoids react with aluminium chloride for quantification and have widely been accepted. In summary, 1 g ground material was mixed with 50 mL methanol (90%), soaked for 3 h, and subjected to ultrasonic-assisted extraction. Following ultrasonic treatment, centrifugation was carried out at 11,000× *g* for 10 min and the supernatant was obtained. The extraction process was repeated three times, and the collected solution was combined. Filtration of the supernatant was carried out using Whatmann filter paper, and the resulting extract was stored for use in TFC determination.

#### 4.3.2. Determination of TFC

The total flavonoids content (TFC) was determined using the colorimetric method as described by Zuo et al. [67], with some modifications. Briefly, 80 µL of twice diluted crude extract was mixed with NaNO_2_ (80 µL 5% W/V) solution and then shaken for 6 min. AlCl_3_ (80 µL 10% W/V) was added and allowed to stand for 6 min. Then, NaOH (400 µL 4% W/V) solution was added and allowed to react for 15 min. Afterward, the absorbance of the reaction mixture was read at 510 nm with UV/VIS spectrophotometer with methanol used as the blank. TFC of each sample was determined from a rutin standard curve, and the results were expressed in mg of rutin in 1 g dry material. 

### 4.4. Transcriptomics

#### 4.4.1. Total RNA Extraction, RNA Library Construction, and Sequencing

Using the three tissues from *C. rotundifolia*, RNA was separately extracted using a general plant total RNA extraction kit (BioTeke Corporation., Ltd. cat. NO RP3301, Wuxi, China) following the methods by Chomczynski and Sacchi [68]. The extracted RNA samples were quantified using a NanoDrop^TM^ One^C^ spectrophotometer (Thermo Fisher Scientific Inc. Waltham, USA.) and the quality was confirmed by agarose gel electrophoresis. The RNA integrity number (RIN) for the samples was 7.8 for stem and leaf and 7.6 for root tissue. To generate libraries for Illumina sequencing, Oligo(dT)-attached magnetic beads were used to purify mRNA. Purified mRNA was fragmented into small pieces with fragment buffer at an appropriate temperature. First-strand cDNA was generated using random hexamer-primed reverse transcription, followed by second-strand cDNA synthesis. Afterward, A-Tailing Mix and RNA Index adapters were added by incubating to end repair. The cDNA fragments obtained from the previous step were amplified by PCR, and products purified by Ampure XP Beads, then dissolved in EB solution. The product was validated on the Agilent Technologies 2100 bioanalyzer for quality control. The double-stranded PCR products from the previous step were heat-denatured and circularized by the splint oligo sequence to get the final library. The single-strand circular DNA (ssCir DNA) was formatted as the final library. The final library was amplified with phi29 to make a DNA nanoball (DNB) which had more than 300 copies of one molecular. DNBs were loaded into the patterned nanoarray and paired-end 150 base reads were generated on the MGISEQ-2000 platform (BGI-Shenzhen, China). Raw data was submitted to the NCBI sequence read archive (SRA) database (https://dataview.ncbi.nlm.nih.gov/object/PRJNA728209?reviewer=oeqs1aq76g906vji4doppnf225 (Accessed on: 25 October 2021)).

#### 4.4.2. RNA-Seq Data Analysis and Functional Annotation

Raw sequencing data were first filtered by Trimmomatic (version 0.38), low-quality reads were discarded, and the reads contaminated with adaptor sequences were trimmed. Clean reads were then mapped to the reference genome of *C. rotundifolia* (deposited at the Genomic Data Center, BioProject PRJCA005006) using TopHat software (version 2.1.1) with default parameters. Reads mapped to the exon regions of each gene were counted by Cufflinks (version 2.2.1) and then fragments per kilobase per million fragments (FPKM) were calculated. 

Genes differentially expressed between groups were identified using the Cuffdiff (version 2.2.1). A *p*-value cutoff of 0.05 and a fold-change cutoff of 2 were used to judge the statistical significance of gene expression differences. Gene ontology (GO) (https://wego.genomics.cn/ (Accessed on: 25 October 2021)), Clusters of Orthologous Groups of proteins (COG), and Kyoto encyclopedia of genes and genomes (KEGG) annotation for expressed genes were implemented by eggNOG software. The visualization of GO annotation was by WEGO 2.0 (https://wego.genomics.cn/ (Accessed on: 25 October 2021)) while COG functional groups were visualized using GraphPad Prism version 8.0.0 for Windows (GraphPad Software, San Diego, CA, USA).

#### 4.4.3. RNA-Seq Data Validation Using qPCR

Total RNA was extracted using a general plant total RNA extraction kit (BioTeke Corporation Co., Ltd. Cat. # RP3301, Wuxi, China). DNA was removed from the RNA sample using the RQ1 RNase-Free DNase kit (Promega Cat. # M6101, Beijing, China) following the manufacturer’s instructions. First-strand cDNA synthesis was carried out using HiScript^®^ III 1st Strand cDNA Synthesis Kit (Vazyme Biotech Co., Ltd. Cat # R312-01, Nanjing, China) following the manufacturer’s instructions in a 20 µL total reaction volume. The qPCR analysis was performed using a 7500 Fast Real-Time PCR system (Applied Biosystems, MA, USA) in a total of 10 µL reaction volume using ChamQ universal SYBR qPCR Master Mix (Vazyme Biotech Co., Ltd., Vazyme code: Q711-02, Nanjing, China). The amplification conditions were 50 °C for 20 s, 95 °C for 10 min, 40 cycles of 95 °C for 15 s, and 60 °C for 1 min. Three biological and two technical replicates per sample were carried out, and Actin was used as the internal standard. The Ct value was determined using the instrument’s software, and the relative quantification of gene expression was monitored after normalization using Actin. The relative transcription levels were calculated using the ΔΔCt method [69], and leaf was considered as the control tissue for normalized relative expression. 

## 5. Conclusions

In this study through metabolomic approaches, 194 flavonoids were tentatively identified in *C. rotundifolia*. Additionally, 18,427 expressed genes from leaf, stem, and root transcriptome were identified and mapped onto the *C. rotundifolia* genome. Regulatory mechanisms involved in flavonoids accumulation were also explored across the tissues through comparative analysis of metabolite accumulation and the expression profile for flavonoids-related biosynthesis genes. In addition to the enzymes involved in the central flavonoids biosynthesis pathway, which we suggest may have influenced flavonoids accumulation in different tissues of *C. rotundifolia*, we hypothesize that transcription factors may also have contributed to the variable flavonoid concentration across tissues. To our knowledge, no prior report has been made on differences in flavonoids accumulation among different tissues of *C. rotundifolia* that are used variably for medicinal value. Our study provides valuable information about flavonoids metabolites and contributes to molecular research in *Cissus rotundifolia*, as well as other members in this genus and facilitates exploration of their medicinal uses.

## Figures and Tables

**Table 1:** A list of 30 flavonoids tentatively identified in *C. rotundifolia* tissues.

Metabolite Name	Precursor Ion (Q1) (Da)	Product Ion (Q3) (Da)	Retention Time (min)	Main Fragments (Da)
Diosmetin (5,7,3′-Trihydroxy-4′-methoxyflavone)	299.06	256.04	6.1	256.04, 284.03, 299.05, 299.13, 299.06
Quercetin-3-o-galactoside (Hyperin)	463.1	300.03	4.2	300.03, 301.03, 463.09
Catechin gallate	441.3	289.08	4.2	124.02, 125.03, 169.02, 193.01, 203.07, 245.08, 289.08, 331.05, 441.08
Kaempferol-3-o-rhamnoside (Afzelin)(Kaempferin)	431	284.04	5	229.05, 227.04, 255.03, 284.04, 285.05, 431.11
Epiafzelechin	275.1	139.04	4.2	107.05, 111.04, 121.06, 139.04, 149.06, 145.06, 173.06, 191.07, 275.09
Delphinidin	303.05	137.02	4.8	137.02, 153.02, 165.02, 129.05, 257.04
Isoorientin-7-o-(6″-p-coumaroyl)glucoside	757.2	637.16	4.2	147.04, 291.08, 309.09, 329.07, 353.07, 431.1, 449.11, 577.13, 637.15, 757.20
Kaempferol-3-o-rutinoside(Nicotiflorin)	593.16	285	4.3	285.04, 593.15
Kaempferol-3-arabinopyranoside	419.1	133.05	5	133.05, 287.06
Epicatechin	291	123.05	3.8	119.05, 123.05, 139.04, 147.04, 165.06, 161.06, 179.07, 207.07, 291.09
Isohemiphloin	433.12	313.07	4.2	125.02, 152.99, 211.06, 271.06, 331.07, 343.08, 359.15, 433.23, 433.11, 433.2
Calycosin	285	225.06	5.5	225.09, 242.06, 269.04, 270.05, 285.08
5-Hydroxy-6,7,8,3′,4′-pentamethoxyflavone	389.1	359.08	7.2	341.08, 359.07, 389.12
Pratensein	301.07	269.04	6.4	167.04, 181.07, 258.05, 269.08, 286.05, 301.07
Aureusidin	287.05	153.02	5.6	153.02, 287.06
5-Hydroxyauranetin	389.1	359.07	7	341.06, 359.07, 389.12
Epigallocatechin	305	219.07	3	125.02, 137.02, 167.03, 165.02, 179.03, 219.07, 221.05, 305.07
Gallocatechin	307	163.04	2.8	123.04, 139.04, 163.04, 177.05, 195.06, 233.06
Kaempferol (3,5,7,4′-Tetrahydroxyflavone)	285.05	229.05	6.2	151.00, 185.06, 211.04, 229.05,
Naringenin-7-o-glucoside (Prunin)	433	151	4.8	119.05, 151.00, 177.02, 255.03, 271.06, 284.03, 301.03, 417.08, 433.11
Luteolin-8-c-glucoside (Orientin)	449.1	329.07	4	287.06, 299.06, 329.07, 353.07, 383.08, 413.09, 431.10, 449.11
Avicularin(Quercetin-3-o-α-L-arabinofuranoside)	435.08	303	4.5	303.05, 257.04, 229.05
Quercetin-3-o-arabinoside (Guaijaverin)	433.08	255.03	4.7	151.00, 179.00, 255.03, 271.02, 300.03, 301.04, 433.08
Isoorientin-7-o-glucoside	611.1	329.07	3.5	299.06, 319.04, 329.07, 383.08, 431.10, 449.11, 465.10, 611.15
Vitexin-7-o-(6″-p-coumaroyl)glucoside	741.2	415.1	4.4	147.04, 309.11, 313.07, 337.07, 415.10, 433.11, 741.20
Luteolin-7-o-rutinoside	595.16	287.05	4.3	287.06, 449.11
Phloretin	273.08	123.04	6	119.05, 123.04, 167.03, 273.08
Kaempferol-3,7-o-dirhamnoside (Kaempferitrin)	579.2	433.11	4.3	287.05, 433.11
Quercetin	303.04	229.05	5.6	153.02, 165.02, 229.05, 257.04, 285.04
Kaempferol-3-o-arabinoside (Juglanin)	417.1	284.03	4.9	227.04, 255.03, 284.04, 285.04, 417.09

**Table 2:** Summary of annotated central genes involved in flavonoids biosynthesis in *C. rotundifolia* and *D. cambodiana *as previously reported [40].

Enzyme	Gene Designation	No. of Annotated Sequences (*C. rotundifolia*)	No. of Annotated Sequences (*D. cambodiana*)
**Phenylalanine ammonia-lyase**	* PAL *	7	6
**Cinnamic acid 4-hydroxylase**	* C4H *	4	1
**4-Coumaric acid: CoA ligase**	* 4CL *	8	18
**Chalcone synthase**	* CHS *	1	10
**Chalcone isomerase**	* CHI *	7	6
**Cinnamoyl-CoA**	* CCR *	2	-
**Flavanone 3-hydroxylase**	* F3H *	1	7
**Flavonoid 3′,5′-hydroxylase**	* F3′5′H *	1	-
**Shikimate-o-hydroxycinnamoyltransferase**	* HCT *	6	-
**Flavonol synthase**	* FLS *	5	10
**Dihydroflavanol 4-reductase**	* DFR *	1	16
**Leucoanthocyanidin dioxygenase/anthocyanidin synthase**	* LDOX/ANS *	2	-
**Anthocyanidin reductase**	* ANR *	1	-
**Leucoanthocyanidin reductase**	* LAR *	6	1

## Data Availability

Transcriptome raw data has been deposited in the SRA database (to be released; https://dataview.ncbi.nlm.nih.gov/object/PRJNA728209?reviewer=oeqs1aq76g906vji4doppnf225 (Accessed on: 25 October 2021)). The genome data used in this study were deposited on National Genomics Data Center (NGDC, https://ngdc.cncb.ac.cn/ (Accessed on: 25 October 2021)) with the project numbers PRJCA005006. All data can be obtained from the corresponding author upon reasonable request.

## Supplementary Materials

S1: The following are available online at https://www.mdpi.com/article/10.3390/metabo11110741/s1. Table S1: Flavonoids metabolites in the tissues of *C. rotundifolia. *The table represents the compound identification details including retention time, molecular ions and fragmentation. Differentially accumulated metabolites are also included, Table S2: GO details for major sub-ontologies for expressed genes in *C. rotundifolia* transcriptome, Table S3: KEGG details for expressed genes for *C. rotundifolia*, Table S4: qPCR primers. The primers were designed using NCBI Primer-BLAST. *PAL*, phenylalanine ammonialyase; *C4H*, Cinnamate 4-hydroxylase; *CHS*, chalcone synthase; *CHI*, chalcone isomerase; *F3H*, flavanone 3-hydroxylase; *4CL*, 4-Coumaric acid: CoA ligase; *FLS*, flavonol synthase. Table S5: Statistics for GO annotation of DEGs identified in *C.* rotundifolia, Figure S1: Overall qualitative and quantitative analysis of metabolomic data. PCA analysis for the 3 *C*. *rotundifolia* tissues. The x-axis represents the first primary principal component while the y-axis represents the second primary principal component. Distinct patterns were observed within different tissues, Figure S2: Differentially accumulated metabolites (DAMs) among leaf, stem, and root for *C. rotundifolia*. (a) Volcano plot representing DAMs in L-vs-S; (b) DAMs in R-vs-L; (c) DAMs in R-vs-S. The spots represent the DAMs; red for up-accumulated, green for down-accumulated while black for those not significantly changed, Figure S3: Functional annotation of *C*. *rotundifolia* transcriptome. (a) Gene Ontology (GO) Classification. The three main categories were identified (cellular components, molecular function, and biological process). The left y-axis represents the gene percentage while the right y-axis indicates the number of genes in the categories. (b) COG terms. The genes were classified into 25 functional categories. The letters represent respective functional categories, Figure S4: RNA-seq validation by qPCR. The histograms indicate the qPCR results for 12 selected genes involved in flavonoid biosynthesis in 3 organs of *C. rotundifolia*. The error bars represent the mean SD of three biological replicates, Figure S5: Transcription factors expression profile. The expression major transcription factor families involved in flavonoid biosynthesis were analysed. (a) bHLH transcription factors, (b) MYB transcription factors, (c) WD40 transcription factors, Figure S6: Classification of *C. rotundifolia* identified DEGs to KEGG pathways. (a) Leaf-vs-Stem; (b); Root-vs-Leaf (c) Root-vs-Stem.
