# Supplementary material for: Characterization of Flavonoids and Transcripts Involved in Their Biosynthesis in Different Organs of Cissus rotundifolia Lam
Source: Metabolites. 2021 Oct 28;11(11):741. doi: 10.3390/metabo11110741 (PMC8621200; doi:10.3390/metabo11110741)
Supplement: Supplementary file 1 [file metabolites-11-00741-s001.zip › Supplementary files/Supplementary figures and tables .pdf]

Gichuki et al. 2021. Characterization of Flavonoids and Transcripts Involved in their Biosynthesis in Different Organs of Medicinal Plant *Cissus rotundifolia* Lam.

Supplementary figures:

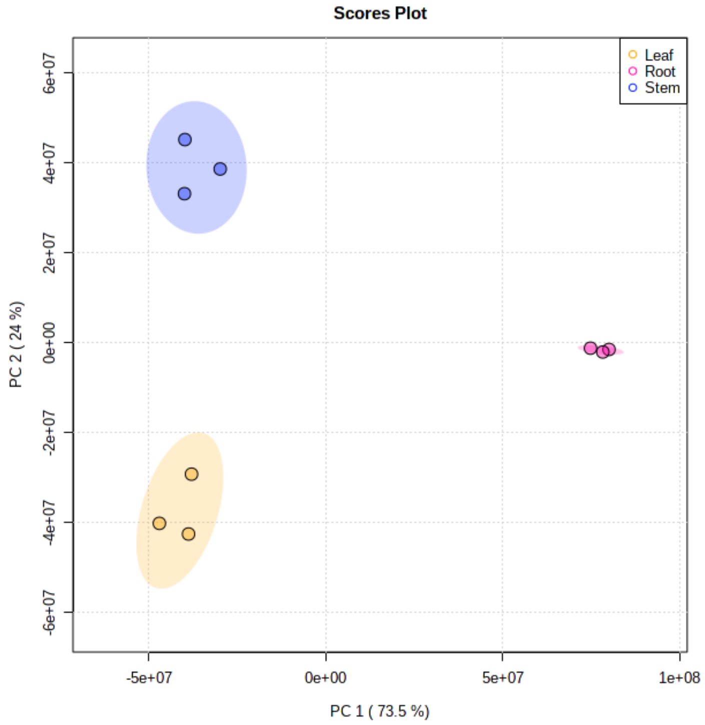

Supplementary Figure S1 Overall qualitative and quantitative analysis of metabolomic data. PCA analysis for the 3 *C. rotundifolia* tissues. The x-axis represents the first primary principal component while the y-axis represents the second primary principal component. Distinct patterns were observed within different tissues.

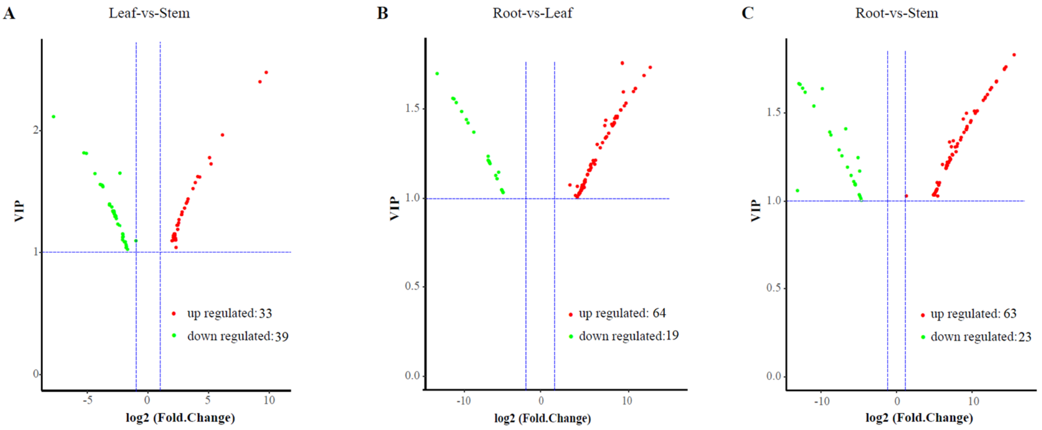

Supplementary Figure S2: Differentially accumulated metabolites (DAMs) among leaf, stem, and root for *C. rotundifolia*. a) Volcano plot representing DAMs in L-vs-S; b) DAMs in R-vs-L; c) DAMs in R-vs-S. The spots represent the DAMs; red for up-accumulated, green for down-accumulated while black for those not significantly changed.

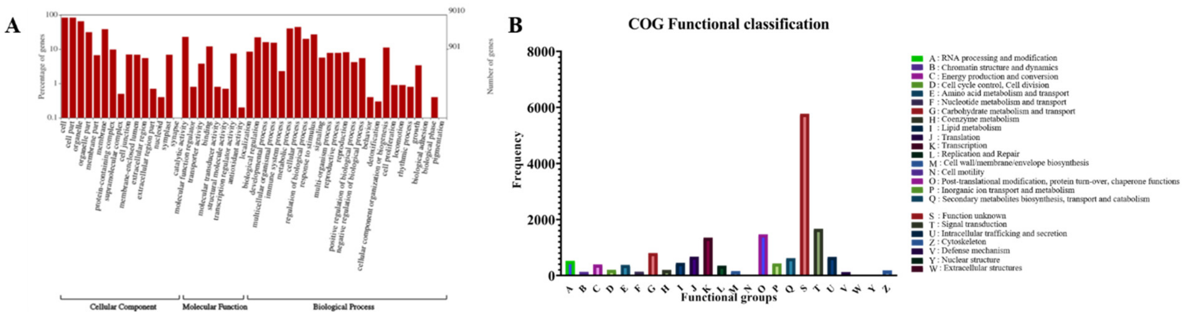

Supplementary Figure S3 function annotation of *C. rotundifolia* transcriptome. a) Gene Ontology (GO) Classification. The three main categories were identified (cellular components, molecular function, and biological process). The left y-axis represents the gene percentage while the right y-axis indicates the number of genes in the categories. b) COG terms. The genes were classified into 25 functional categories. The letters represent respective functional categories

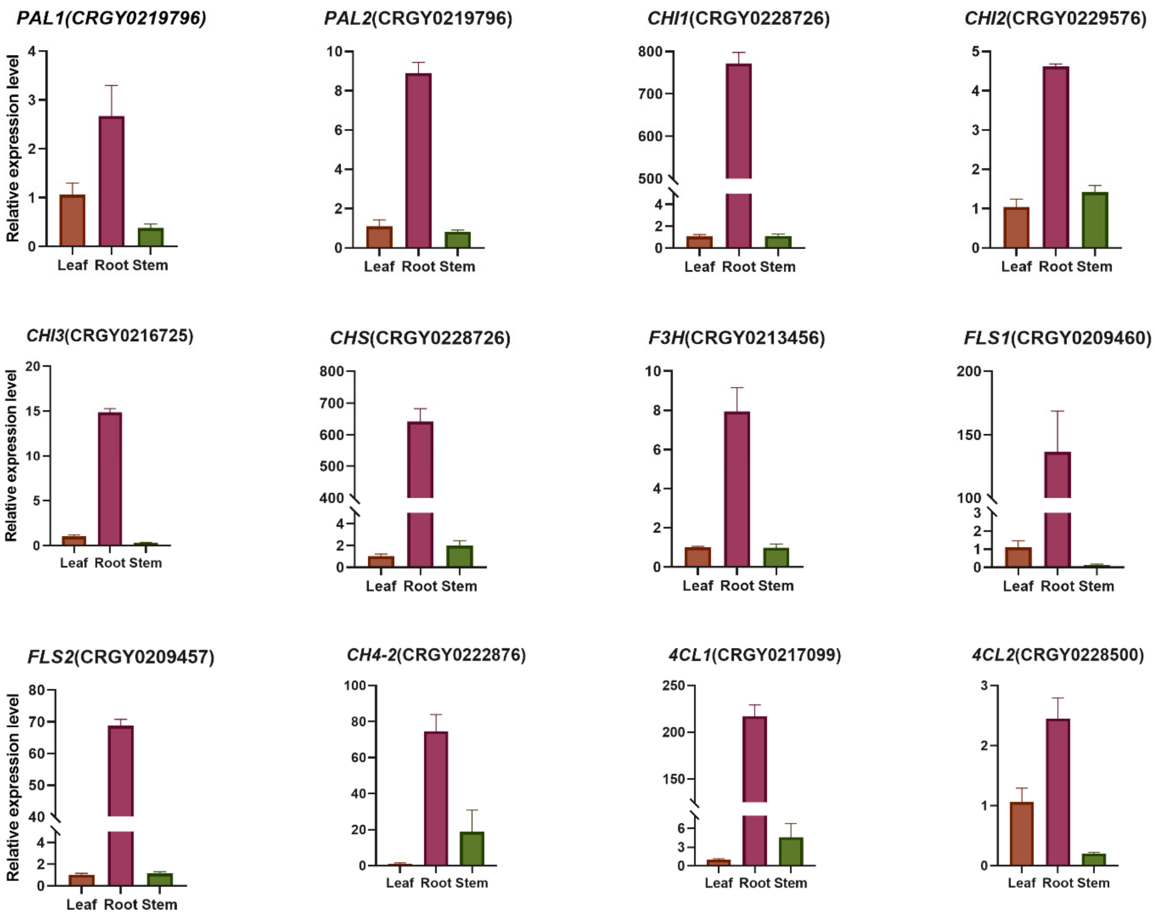

Supplementary Figure S4: RNA-seq validation by qPCR. The histograms indicate the qPCR results for 12 selected genes involved in flavonoid biosynthesis in 3 organs of *C. rotundifolia*. The error bars represent the mean SD of three biological replicates.

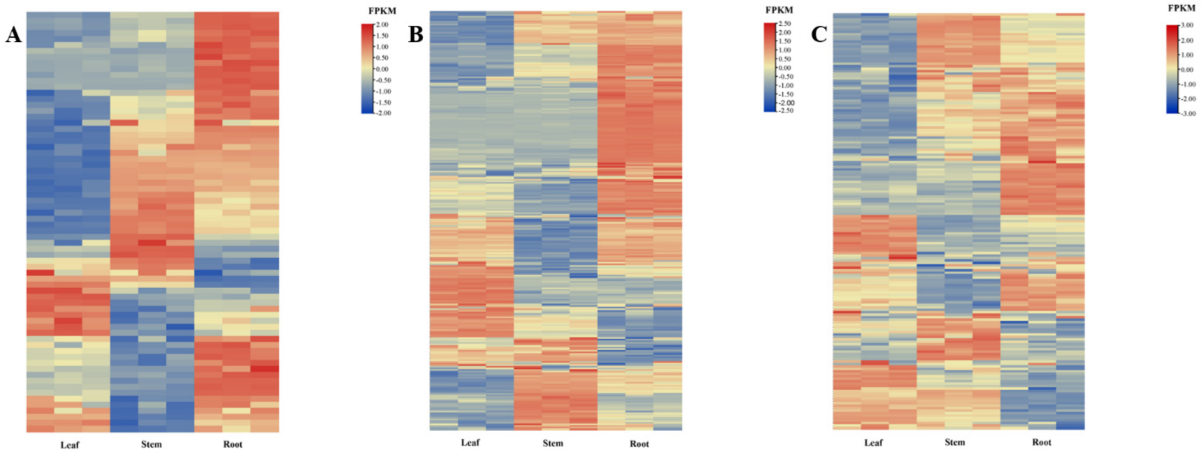

Supplementary Figure S5. Transcription factors expression profile. The expression major transcription factor families involved in flavonoid biosynthesis were analysed. a) bHLH transcription factors, b) MYB transcription factors, c) WD40 transcription factors

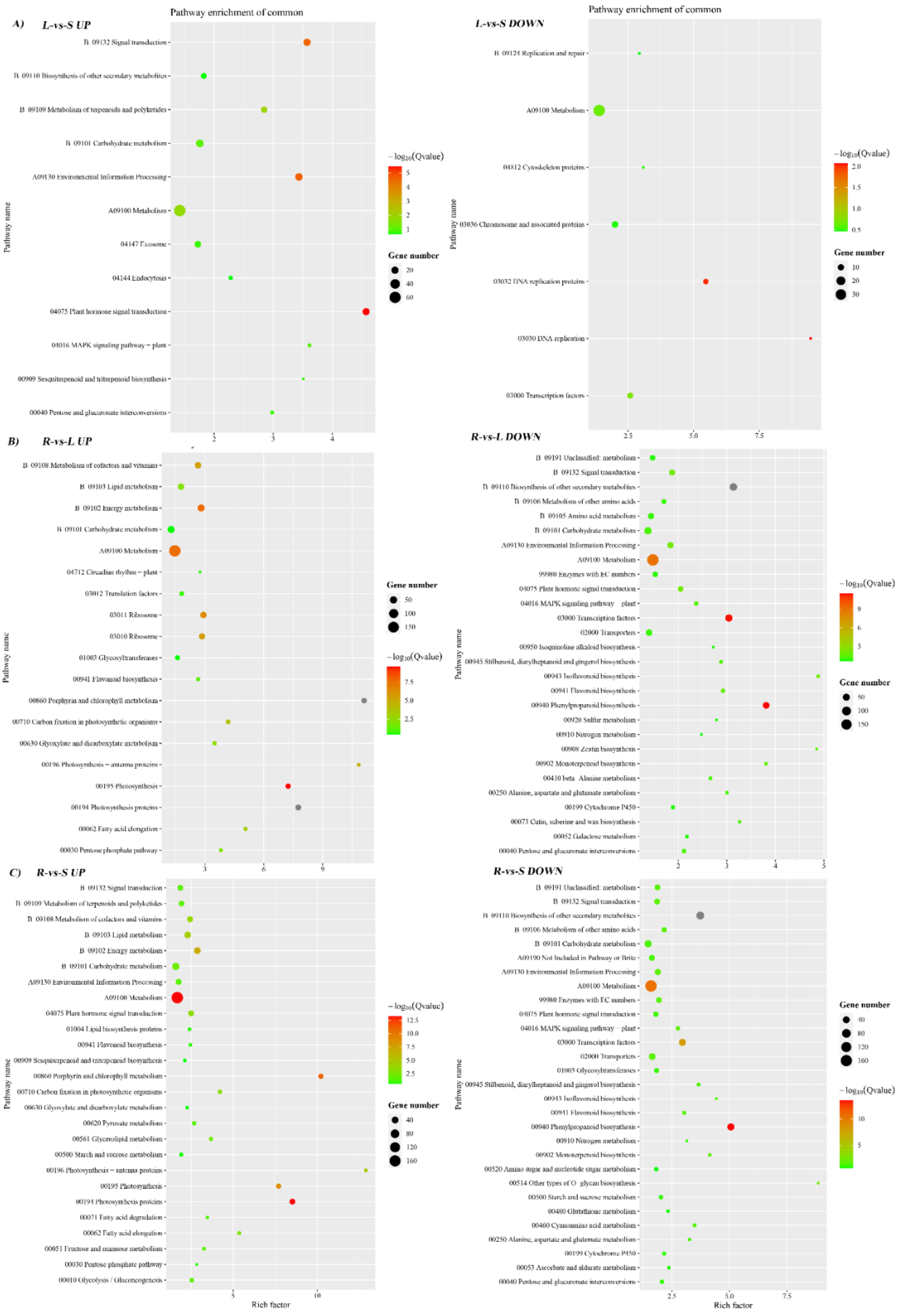

Supplementary Figure S6 : Classification of *C. rotundifolia* identified DEGs to KEGG pathways. a) Leaf-vs-Stem; b); Root-vs-Leaf c) Root-vs-Stem.

Supplementary Tables:

Supplementary Table S1: Summary of *Cissus rotundifolia* sequencing results. The letters represent the sampled organs; Stem-S, L-Leaf, and R for root. The numbers indicate the replicates

Sample Name	Clean Reads	Clean Base	Read Length	Q20(%)	GC(%)
S_1	40302432	6.05E+09	150	95.77	46.14
S_2	41267420	6.19E+09	150	95.94	46.2
S_3	40150140	6.02E+09	150	95.75	46.23
L_1	41426178	6.21E+09	150	95.7	46.49
L_2	41483322	6.22E+09	150	95.37	46.63
L_3	40175842	6.03E+09	150	95.73	46.49
R_1	40118942	6.02E+09	150	95.62	44.91
R_2	40851182	6.13E+09	150	95.83	44.96
R_3	41685368	6.25E+09	150	95.93	44.95

Supplementary Table S2: A summary of read mapping of *C. rotundifolia* transcriptome to the genome. The letters represent the sampled organs; Stem-S, L-Leaf, and R for root. The numbers indicate the replicates

Sample	Left/right reads	Left mapped read	Right mapped reads	Left mapped ratio	Right mapped ratio	Mapped ratio
**L_1**	20186661	17780114	17170664	88.1%	85.1%	86.6%
**L_2**	20164465	17355248	16893645	86.1%	83.8%	84.9%
**L_3**	19578017	17202929	16679195	87.9%	85.2%	86.5%
**R_1**	19541178	13937219	13412860	71.3%	68.6%	70.0%
**R_2**	19917380	14250464	13827964	71.5%	69.4%	70.5%
**R_3**	20335517	14500969	14149014	71.3%	69.6%	70.4%
**S_1**	19660872	17301278	16726495	88.0%	85.1%	86.5%
**S_2**	20144022	17762541	17263528	88.2%	85.7%	86.9%
**S_3**	19580911	17198676	16683431	87.8%	85.2%	86.5%

Supplementary Table S3. FPKM for genes selected for RNA-seq validation by qPCR. The FPKM values indicate the average from three biological replicates for the 3 organs.

Gene name	Gene ID	FPKM		
		Leaf	Root	Stem
PAL1	CRGY0200728	0.000	2.023	0.115
PAL2	CRGY0219796	16.478	93.472	7.612
CHI1	CRGY0228726	0.050	17.823	0.076
CHI2	CRGY0229576	8.352	23.710	16.140
CHI3	CRGY0216725	11.866	143.387	28.063
CHS	CRGY0228726	0.050	17.823	0.076
F3H	CRGY0213456	13.058	77.965	21.204
FLS1	CRGY0209460	0.315	227.811	0.544
FLS2	CRGY0209457	0.072	268.336	0.142
C4H	CRGY0222876	0.459	12.078	2.795
4CL1	CRGY0217099	0.197	25.665	0.482
4CL2	CRGY0228500	4.166	32.422	1.797

Supplementary Table S4. **qPCR primers**. The primers were designed using NCBI Primer-BLAST. *PAL*, phenylalanine ammonialyase; *C4H*, Cinnamate 4-hydroxylase; *CHS*, chalcone synthase; *CHI*, chalcone isomerase; *F3H*, flavanone 3-hydroxylase; *4CL*, 4-Coumaric acid: CoA ligase; *FLS*, flavonol synthase.

Gene names*	Primer sequences (5'-3’)	Product sizes (bp)
PAL1	F: AGAGGTTGAGAGCGCCAGAC<br>R: AGGGAAGAGCTGGGAACAGC	96
PAL2	F: TTCGCCTACATCGACGACCC<br>R: GATCGAGGTGCTGACGCTCT	120
CHI1	F: TGGTGGCCCAGCAATTCTTGAT<br>R: CATGCACTTGACATGTTCCCGA	117
CHI2	F: TTTGAGAAATCCCTTCGTGCCC<br>R: TGTTGTCCCCGCAGGTAATGG	117
CHI3	F: AGGAAGAGGAAGAAGCCTTGGAG<br>R: AGCAGTGCACGAAGTTGCAG	107
CHS	F: GCCCAGCAATTCTTGATGCAGTT<br>R: ATGCACTTGACATGTTCCCGA	100
F3H	F: GCTTGAGGAGCCCATCACCT<br>R: TTGGCCTGTTGTTCCTTGGC	102
FLS1	F: AGTCTTCTGTGCACCACCCC<br>R: GCGGTACTCAGCGAAGGTTT	106
FLS2	F: AACTGGCGCTCGGAGTTGAA<br>R: CAAGTACTCGACGGCGACCC	119
C4H	F: TGGGAAGGAGGAGTTGTCCG<br>R: CCGGAGCCCTCATGTCAAAG	96
4CL1	F: CCGATCTCACCGAACAAGCAC<br>R: TTTGCCAGCAGTGGACCTTG	113
4CL2	F: GCTCTTTTGGTCAGCCACCC<br>R: ACCACAAAAGCAACGGGGAC	92
Actin	F: CACCTGCAGCCAACTTTCACC<br>R: TGGAAGCCGAAGGAAGTCGT	100

Supplementary Table S5: Statistics for GO annotation of DEGs identified in *C. rotundifolia*

GO terms	Root vs Leaf	Root vs Stem	Stem vs Leaf
**Biological Process**	2314	1374	1904
**Cellular component**	3491	2102	2822
**Molecular function**	1640	953	1379
